# Activity of ceftolozane/tazobactam and comparators against gram-negative bacilli: Results from the Study for Monitoring Antimicrobial Resistance Trends (SMART – Brazil), 2018‒2021

**DOI:** 10.1016/j.bjid.2024.104497

**Published:** 2024-12-12

**Authors:** Amanda Azevedo Bittencourt, Vinicius Lima Faustino, Paula de Mendonça Batista, Lays Paulino Leonel, Marina Della Negra de Paula, Thales José Polis

**Affiliations:** a Global Medical & Scientific Affairs (GMSA), MSD Brazil, São Paulo, SP, Brazil; b Real World Evidence, IQVIA Brazil, São Paulo, SP, Brazil

**Keywords:** Antibiotics, Bacterial infections, Gram-negative bacilli (GNB), Surveillance

## Abstract

Increased spread of antimicrobial resistance by Gram-Negative Bacilli (GNB) poses a global challenge, with exacerbated burden post-pandemic. The aim of this study was to investigate the *in vitro* activity of ceftolozane/tazobactam and its comparators against the frequently identified GNB isolated from patients admitted to Brazilian medical sites between the year 2018‒2019 and 2020‒2021. The impact of pandemic on antimicrobial resistance and presence of β-lactamase genes were also evaluated. Antimicrobial susceptibility testing and molecular characterization of ß-lactamase encoding genes using Polymerase Chain Reaction (PCR) and DNA sequencing were carried out from GNB isolated mostly from intra-abdominal, respiratory, and urinary tract infections and interpreted following BrCAST/EUCAST guidelines. A total of 3994 GNB isolates were evaluated which mostly included *E. coli, K. pneumoniae* and *P. aeruginosa.* Ceftolozane/tazobactam remained highly active against *E. coli* isolates during both 2018‒2019 (96.0 %) and 2020‒2021 (98.5 %). Among *K. pneumoniae*, ceftolozane/tazobactam (47.6 % and 43.0 % susceptible during 2018‒2019 and 2020‒2021, respectively) showed poor activity due to *bla*_KPC-2_. Colistin and ceftolozane/tazobactam were the most active β-lactam agents tested against *P. aeruginosa* in 2018‒2019 (99.3 % and 88.8 %) and 2020‒2021 (100 % and 92.8 %), including ceftazidime and meropenem resistant isolates. β-lactamase encoding gene characterization was carried out and both carbapenemases and Extended-Spectrum β-Lactamase (ESBL) producers were found in *E. coli, K. pneumoniae* and *P. aeruginosa* isolates. Ceftolozane/tazobactam documented remarkable *in vitro* activity against *E. coli and P. aeruginosa* isolates in Brazil, both pre- and post-pandemic periods and could constitute an effective therapeutic option for the treatment of urinary tract infections, intra-abdominal infections, and respiratory tract infections.

## Introduction

Antimicrobial Resistance (AMR) is one of the leading global threats. It occurs when changes in bacteria, viruses, fungi and parasites make existing antibiotics ineffective or less effective, increasing the burden on society. The World Health Organization (WHO) emphasizes the need to have a global coordinated action to prevent further spread of AMR.^1^ According to the 2016 AMR review, it was projected that around 10 million individuals might die annually due to AMR by the year 2050.^2^

The rise in bacterial resistance has led to a significant threat posed by Gram-Negative Bacilli (GNB) with Multidrug Resistance (MDR). This poses a challenge to the medical and scientific community as there are limited treatment options available to control infections caused by bacteria such as *K. pneumoniae, P. aeruginosa* and *E. coli*.^3^ As a result of the increased prevalence of Extended-Spectrum β-Lactamase (ESBL) production among GNB, carbapenem antibiotics have been utilized extensively.^4^ In low middle income countries, including Brazil, resistance to carbapenems has developed due to their increased use.^4^, ^5^, ^6^

In Brazil, serious hospital-acquired infections have been linked to GNB exhibiting resistance to various antimicrobial agents.^7^ The pandemic caused by the Coronavirus Disease (COVID-19) has significantly intensified the challenge of AMR. This escalation can be attributed to the surge in infection rates, which has led to an increase in hospital admissions and the use of invasive medical devices. Consequently, this has resulted in prolonged hospital stays and a higher mortality rate.^8^, ^9^, ^10^ Furthermore, there has been a noticeable rise in carbapenem resistance during the pandemic period.^4^^,^^11^^,^^12^

Health authorities in many countries have recommended the use of alternative antibiotics and different combinations of medications to reduce the further spread of resistance.^13^ Novel β-Lactam/β-Lactamase Inhibitors (BL/BLIs) including ceftolozane/tazobactam, ceftazidime/avibactam, and imipenem/relebactam have emerged as salvage therapies for infections due to pathogens that are resistant to most antibiotics.^14^^,^^15^

Ceftolozane/tazobactam is comparatively a newer antimicrobial that was approved by the United States Food and Drug Administration in 2014 for the treatment of complicated Intra-Abdominal Infections (cIAI) and complicated Urinary Tract Infections (cUTI), including pyelonephritis at a dosage of 1.5 g, three times a day and hospital-acquired/ventilator-associated bacterial pneumonia (HABP/VABP) at a dosage of 3 g every 8 h.^16^, ^17^, ^18^ It was approved in Brazil by the national health regulatory agency in Portuguese *Agência Nacional de Vigilância Sanitária* (ANVISA) for cUTI and cIAI in 2018 and for HABP/VABP in 2020.^19^, ^20^, ^21^ The drug combines the new cephalosporin ceftolozane having higher affinity for penicillin-binding proteins compared with other β-lactam agents, high stability against amp-C type β-lactamases, with tazobactam providing increased activity against organisms producing ESBL.^17^

Surveillance data at the national level is necessary, along with the establishment of standardized dosage regimens for the utilization of ceftolozane/tazobactam. This is particularly important for patients suffering from severe respiratory infection and hospital-acquired or ventilator-associated bacterial pneumonia (HABP/VABP) who are critically ill.^14^^,^^20^, ^21^, ^22^

The Study for Monitoring Antimicrobial Resistance Trends (SMART) program has generated data on the frequency of antimicrobial susceptibility of GNB associated with Urinary Tract Infections (UTI), Intra-Abdominal Infections (IAI) and Respiratory Tract Infections (RTI), which helps to delineate the changes in the epidemiology of gram-negative infections over time.^19^^,^^23^

The principal aim of this study was to determine the frequency of pathogens and *in vitro* activity of ceftolozane/tazobactam and its comparators against the frequently identified GNB isolated from patients admitted to medical study sites across Brazil between the years 2018‒2019 and 2020‒2021. The study was conducted in two time periods to assess the impact of COVID-19 on AMR.

## Methods

### Bacterial isolates

Non-duplicate GNB isolates were collected from ten study sites across six Brazilian cities: Belo Horizonte (one), Curitiba (one), Recife (one), Rio de Janeiro (two), Salvador (one) and São Paulo (four), from 2018‒2021, as part of the SMART surveillance program.

GNB were identified at the species level at the respective participant medical sites and shipped to a central microbiology laboratory (International Health Management Associates, IHMA, Schaumburg, IL, USA), where confirmation of bacterial species, antimicrobial susceptibility testing, and molecular characterization of β-lactamase encoding genes were carried out. Bacterial identification at the species level was confirmed for all isolates using MALDI-TOF spectrometry (Bruker Daltonics, Billerica, MA, USA).

### Susceptibility testing

Antimicrobial susceptibility testing for amikacin, aztreonam, cefepime, cefotaxime, ceftazidime, ceftolozane/tazobactam, ceftriaxone, ciprofloxacin, colistin, ertapenem, imipenem, meropenem, and piperacillin/tazobactam was determined by the Clinical & Laboratory Standards Institute (CLSI) reference broth microdilution method^24^ using broth microdilution panels prepared at IHMA and were interpreted following BrCAST/EUCAST guidelines.^25^^,^^26^ Quality control (QC) of broth microdilution panels followed CLSI guidelines using the ATCC strains: *E. coli* ATCC 25,922, *P. aeruginosa* ATCC 27,853, *K. pneumoniae* ATCC 700,603 and *K. pneumoniae* BAA 2814, with corresponding QC values within the specified acceptable ranges. *E. coli* and *K. pneumoniae* isolates with Minimal Inhibitory Concentrations (MIC) ≥2 µg/mL for ceftazidime, ceftriaxone, or aztreonam were screened as “ESBL phenotype”. Enterobacterales with MIC ≥4 µg/mL for imipenem and/or meropenem were defined as carbapenem resistant. *P. aeruginosa* isolates having MICs > 8 µg/mL and > 2 µg/mL were classified as not susceptible to ceftazidime and meropenem, respectively.

### Molecular characterization of β-lactamase encoding genes

Isolates meeting the following phenotypic criteria were screened for β-lactamase genes: non-Morganellaceae Enterobacterales (NME) isolates (excluding *Serratia* spp.) testing with imipenem or imipenem/relebactam MIC values of ≥ 2 mg/L; P*. aeruginosa* isolates testing with imipenem or imipenem/relebactam MIC values of ≥ 4 mg/L; NME and *Serratia* spp. isolates testing with ertapenem MIC values of ≥ 1 mg/L collected in 2018 only; isolates of *Serratia* spp. testing with imipenem MIC values of ≥ 4 mg/L collected in 2018; and Enterobacterales and *P. aeruginosa* isolates testing with ceftolozane/tazobactam MIC values of ≥ 4 mg/L and ≥ 8 mg/L, respectively. Previously published multiplex PCR assays were used to screen for the following β-lactamase genes (bla): ESBLs (CTX-M, GES, PER, SHV, TEM, VEB); acquired AmpC β-lactamases (ACC, ACT, CMY, DHA, FOX, MIR, MOX); serine carbapenemases (GES, KPC, OXA-48-like [Enterobacterales], OXA-24-like [*P. aeruginosa*]); and Metallo-β-Lactamases (MBLs) (GIM, IMP, NDM, SPM, VIM).^23^ All detected acquired β-lactamases genes were re-amplified using gene-flanking primers and sequenced in full (Sanger) with the exception that limited sequencing was performed on bla_TEM_ and bla_SHV_ to identify genes encoding bla_TEM-type_ and bla_SHV-type_ enzymes containing amino acid substitutions common to ESBLs (SHV A146 V, G238S, G238A, E240 K; TEM E104 K, R164S, R164C, R164H, G238S). Limited sequencing was also performed on bla_CTX__−__M_ to identify the presence of the D240G substitution in the deduced amino acid sequence associated with increased ceftazidime hydrolysis. For *P. aeruginosa* isolates, collected in 2020 and 2021, characterization was performed using short-read whole-genome sequencing (Illumina Hiseq 2 × 150 bp reads) to a targeted coverage depth of 100×^25^ and analyzed using the CLC Genomics Workbench (Qiagen). The Resfinder database was used to detect β-lactamase genes in whole-genome sequencing assemblies.^26^ Per SMART protocol for Enterobacterales isolates collected in 2021, a representative sample of approximately 95 % of isolates meeting the criteria for molecular characterization were characterized. Per SMART protocol for *P. aeruginosa* isolates collected in 2020 and 2021, a representative sample of approximately 75 % of isolates meeting the criteria for molecular characterization were characterized.

### Data analysis and availability

All data analyses were performed in Excel (Microsoft, Redmond, WA). Data are available on request.

## Results

**Fig. 1:**
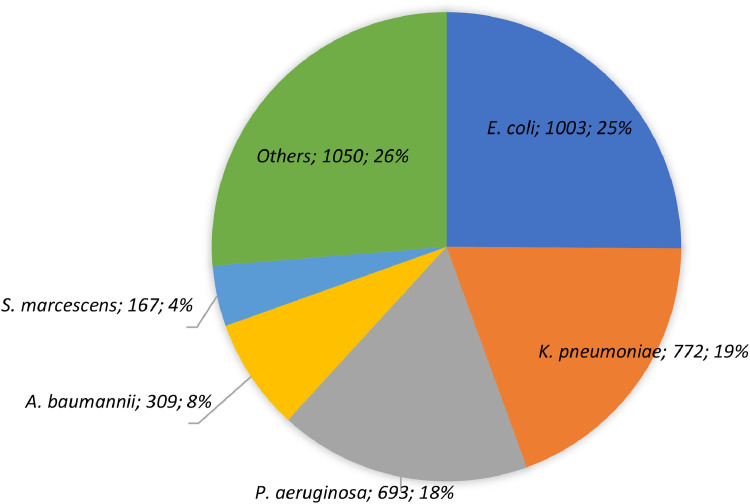
Distribution of isolates according to the bacterial species collected from participating Brazilian medical centers of the SMART Program (Brazil, 2018‒2021).

A total of 3994 GNB isolates were collected from all the study sites between 2018‒2021 [2018, *n* = 754 (18.9 %); 2019, *n* = 982 (24.6 %); 2020, *n* = 951 (23.8 %); 2021, *n* = 1307 (32.7 %)]. More than half of the total isolates were recovered from male patients (*n* = 2190, 54.8 %) aged 50 and above (*n* = 2861, 71.6 %). The most frequent hospital ward was General Medicine (*n* = 1433, 35.9 %), followed by Intensive Care Units (ICUs) (*n* = 1029, 25.8 %). About one-fourth of the isolated species were *E. coli* (*n* = 1003, 25.1 %), while the other commonly isolated species included were *K. pneumoniae* (*n* = 772, 19.3 %) and *P. aeruginosa* (*n* = 693, 17.4 %) (Fig. 1).

**Fig. 2:**
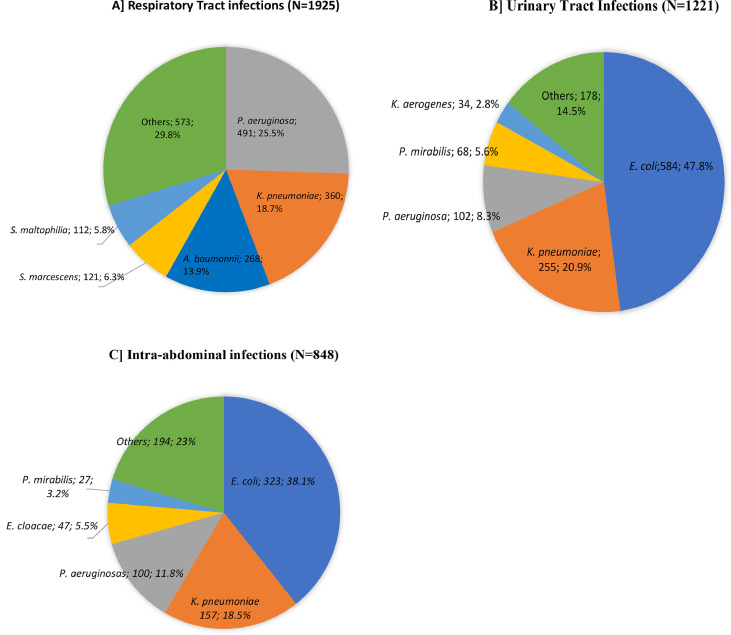
Distribution of bacterial species according to the site of infection (SMART Program – Brazil 2018‒2021).

The distribution of the five most frequent GNB species according to the site of infection is depicted in Fig. 2. Most of the isolated pathogens were obtained from RTI (*n* = 1925, 48.2 %) followed by UTI (*n* = 1221, 30.6 %) and IAI (*n* = 848, 21.2 %). The most commonly isolated pathogens in RTI were *P. aeruginosa* (*n* = 491, 25.5 %), *K. pneumoniae* (*n* = 360, 18.7 %) and *A. baumannii* (*n* = 268, 13.9 %). The most frequent species isolated in UTI were *E. coli* (*n* = 584, 47.8 %), followed by *K. pneumoniae* (*n* = 255, 20.9 %) and *P. aeruginosa* (*n* = 102, 8.3 %). For IAI, the most frequent isolated species were *E. coli* (*n* = 323, 38.1 %), *K. pneumoniae (n* = 157, 18.5 %) and *P. aeruginosa* (*n* = 100, 11.8 %).

### Antimicrobial susceptibility

Antimicrobial susceptibility profiles of the most frequent GNB causing infections are elaborated in Table 1. During the period 2018‒2019, ceftazidime/avibactam (MIC_50/90_, ≤ 0.12/ 0.25 µg/mL, *n* = 532, 100 % susceptible) and imipenem/relebactam (MIC_50/90_, ≤ 0.12/ ≤ 0.25 µg/mL, *n* = 532, 100 % susceptible), were the most active *in vitro* agents tested against the *E. coli* isolates, followed by meropenem (MIC_50/90_, ≤ 0.12/ ≤ 0.12 µg/mL, *n* = 530, 99.6 % susceptible), colistin (MIC_50/90_, ≤ 1/ ≤ 1 µg/mL, *n* = 528, 99.2 % susceptible), imipenem (MIC_50/90_, ≤ 0.12/ ≤ 0.25 µg, *n* = 524, 98.5 % susceptible), and ertapenem (MIC_50/90_, ≤ 0.12/ ≤ 0.12 µg, *n* = 518, 97.3 % susceptible).

**Table 1:** Antimicrobial susceptibility profile of the most frequent GNB causing infections in the Brazilian population.

GNB Antimicrobial Agents (N1, N2)	Broth Microdilution (µg/mL)				EUCAST			
	MIC_50_		MIC_90_		*S*+SIE (%)		R (%)	
	2018‒2019	2020‒2021	2018‒2019	2020‒2021	2018‒2019	2020‒2021	2018‒2019	2020‒2021
***E. coli***								
Amikacin (532, 471)	≤8	≤8	≤8	≤8	95.7 %	97.6 %	4.3 %	2.3 %
Aztreonam (532, 471)	≤1	≤1	>8	>8	79.8 %	82.5 %	20.1 %	17.4 %
Cefepime (532, 471)	≤1	≤1	>16	16	81.0 %	84.7 %	18.9 %	15.2 %
Ceftazidime (532, 471)	≤1	≤1	16	8	81.9 %	86.0 %	18.0 %	14.0 %
Ceftazidime/avibactam (532, 471)	≤0.12	≤0.12	0.25	0.25	100.0 %	99.4 %	0.0 %	0.6 %
Ceftolozane/tazobactam (532, 471)	≤0.12	≤0.12	0.5	0.5	96.0 %	98.5 %	3.9 %	1.4 %
Ceftriaxone (532, 471)	≤1	≤1	>8	>8	75.1 %	75.8 %	24.8 %	24.2 %
Ciprofloxacin (195)	0.5	‒	>2	‒	50.2 %	‒	49.7 %	‒
Colistin (532, 471)	≤1	≤1	≤1	≤1	99.2 %	99.8 %	0.7 %	0.2 %
Ertapenem (532, 471)	≤0.12	≤0.12	≤0.12	≤0.12	97.3 %	97.2 %	2.6 %	2.7 %
Imipenem (532, 471)	≤0.12	0.25	0.25	0.5	98.5 %	98.9 %	1.5 %	1.0 %
Imipenem/relebactam (532, 471)	≤0.12	0.25	0.25	0.25	100.0 %	99.4 %	0.0 %	0.6 %
Meropenem (532, 471)	≤0.12	≤0.12	≤0.12	≤0.12	99.6 %	99.4 %	0.3 %	0.6 %
Piperacillin/tazobactam (532, 471)	≤4	≤4	16	8	88.9 %	92.7 %	11.0 %	7.2 %
***K. pneumoniae***								
Amikacin (319, 453)	≤8	≤8	16	≤8	88.4 %	78.1 %	11.6 %	21.8 %
Aztreonam (319, 453)	>8	>8	>16	>8	39.8 %	31.3 %	60.1 %	68.6 %
Cefepime (319, 453)	>16	>16	>16	>16	42.0 %	32.6 %	57.9 %	67.3 %
Ceftazidime (319, 453)	>16	>16	>16	>16	40.1 %	32.6 %	59.8 %	67.3 %
Ceftazidime/avibactam (319, 453)	0.5	0.5	2	4	96.2 %	91.3 %	3.7 %	8.6 %
Ceftolozane/tazobactam (319, 453)	4	16	>16	>16	47.6 %	43.0 %	52.3 %	56.9 %
Ceftriaxone (319, 453)	>8	>4	>8	>8	36.6 %	28.7 %	63.3 %	71.3 %
Ciprofloxacin (123)	>2	‒	>2	‒	36.5 %	‒	63.4 %	‒
Colistin (319, 453)	≤1	≤1	>4	>4	88.0 %	82.3 %	11.9 %	17.6 %
Ertapenem (319, 453)	0.25	>2	>4	>4	55.4 %	46.5 %	44.5 %	53.4 %
Imipenem (319, 453)	0.5	1	>16	>16	66.4 %	52.3 %	33.5 %	47.6 %
Imipenem/relebactam (319, 453)	0.25	0.25	1	2	95.9 %	90.7 %	4.0 %	9.2 %
Meropenem (319, 453)	≤0.12	1	>16	>16	72.1 %	59.1 %	27.9 %	40.8 %
Piperacillin/tazobactam (319, 453)	64	>64	>64	>64	38.5 %	32.4 %	61.4 %	67.5 %
***P. aeruginosa***								
Amikacin (288, 405)	≤4	≤4	>32	32	87.8 %	89.3 %	12.1 %	10.6 %
Aztreonam (288, 405)	8	8	>16	>16	78.4 %	79.7 %	21.5 %	20.2 %
Cefepime (288, 405)	4	4	32	32	71.8 %	79.0 %	28.1 %	20.9 %
Ceftazidime (288, 405)	4	4	>32	>32	70.8 %	79.2 %	29.1 %	20.7 %
Ceftazidime/avibactam (288, 405)	2	2	16	8	87.5 %	90.4 %	12.5 %	9.6 %
Ceftolozane/tazobactam (288, 405)	0.5	0.5	8	4	88.9 %	92.8 %	11.1 %	7.1 %
Ciprofloxacin (136,0)	≤0.25	‒	>2	‒	61.7 %	‒	38.2 %	‒
Colistin (288, 405)	≤1	1	≤1	1	99.3 %	100.0 %	0.6 %	0.0 %
Imipenem (288, 405)	2	4	32	32	63.8 %	66.9 %	36.1 %	33.0 %
Imipenem/relebactam (288, 405)	1	1	4	4	80.9 %	81.2 %	19.1 %	18.7 %
Meropenem (288, 405)	1	1	32	16	76.3 %	82.7 %	23.6 %	17.2 %
Piperacillin/tazobactam (288, 405)	8	8	>64	>64	65.2 %	73.5 %	34.7 %	26.4 %

GNB, Gram-Negative Bacilli; N1, Number of isolates tested during 2018‒2019; N2, Number of isolates tested during 2020‒2021; EUCAST, European Committee on Antimicrobial Susceptibility Testing; SI +SIE, Susceptible + Susceptible Increased exposure.

Similar results were observed during the 2020‒2021 period, with slightly lower susceptibility percentages for ceftazidime/avibactam (MIC_50/90_, ≤ 0.12/0.25 µg/mL, *n* = 468, 99.4 % susceptible), meropenem (MIC_50/90_, ≤ 0.12/ ≤ 0.12 µg/mL, *n* = 468, 99.4 % susceptible) and imipenem/relebactam (MIC_50/90_, ≤ 0.12/ ≤ 0.25 µg/mL, *n* = 468, 99.4 % susceptible) (Table 1).

In contrast, the lowest susceptibility rates in the period of 2018‒2019 were observed for ciprofloxacin (MIC_50/90_, 0.5/ > 2 µg/mL, *n* = 98, 50.3 % susceptible), followed by ceftriaxone (MIC_50/90_, ≤ 1/ > 8 µg/mL, *n* = 400, 75.2 % susceptible), and aztreonam (MIC_50/90_, ≤ 1/ > 8 µg/mL, *n* = 425, 79.9 % susceptible). For the period 2020‒2021, ciprofloxacin was not included in the panel of antimicrobials used for GNB infections, and ceftriaxone showed lowest susceptibility (MIC_50/90_, ≤ 1/ > 8 µg/mL, *n* = 357, 75.8 % susceptible).

Ceftazidime/avibactam (MIC_50/90_, ≤ 0.12/0.25 µg/mL, *n* = 134) and imipenem/relebactam (MIC_50/90_, ≤ 0.12/0.25 µg/mL, *n* = 134) remained highly active against *E. coli* isolates exhibiting the ESBL phenotype with a susceptibility of 100 % during 2018‒2019 as shown in Table 2. During 2020‒2021 period, colistin (MIC_50/90_, ≤ 1/ ≤ 1 µg/mL, *n* = 113, 99.1 % susceptible) exhibited higher susceptibility against *E. coli* isolates exhibiting the ESBL phenotype followed by ceftazidime/avibactam (MIC_50/90_, ≤ 0.12/0.25 µg/mL, *n* = 111, 97.4 % susceptible), imipenem/relebactam (MIC_50/90_, 0.25/0.25 µg/mL, *n* = 111, 97.4 % susceptible) and meropenem (MIC_50/90_, ≤ 0.12/ ≤ 0.12 µg/mL, *n* = 111, 97.4 % susceptible).

**Table 2:** Susceptibility rates to distinct antimicrobial agents of the frequent pathogens according to the phenotype of resistance in Brazil.

GNB Antimicrobial Agents (N1, N2)	Broth Microdilution (µg/mL)				EUCAST			
	MIC_50_		MIC_90_		*S*+SIE (%)		R (%)	
	2018‒2019	2020–2021	2018–2019	2020–2021	2018–2019	2020–2021	2018–2019	2020–2021
**ESBL - producing *E. coli***								
Amikacin (134, 114)	≤8	≤8	16	≤8	88.0 %	94.7 %	11.9 %	5.2 %
Aztreonam (134, 114)	>8	8	>16	>8	20.1 %	28.0 %	79.8 %	71.9 %
Cefepime (134, 114	16	8	>16	>16	24.6 %	36.8 %	75.%	63.1 %
Ceftazidime (134, 114)	8	8	>16	>16	28.3 %	42.1 %	71.6 %	57.8 %
Ceftazidime/avibactam (134, 114)	≤0.12	≤0.12	0.25	0.25	100.0 %	97.4 %	0.0 %	2.6 %
Ceftolozane/tazobactam (134, 114)	0.5	0.25	8	1	84.3 %	93.8 %	15.6 %	6.1 %
Ceftriaxone (134, 114)	>8	>8	>8	>8	1.4 %	0.0 %	98.5 %	100.0 %
Ciprofloxacin (52, 0)	>2	‒	>2	‒	15.3 %	‒	84.6 %	‒
Colistin (134, 114)	≤1	≤1	≤1	≤1	97.7 %	99.1 %	2.2 %	0.8 %
Ertapenem (134, 114)	≤0.12	≤0.12	2	0.25	89.5 %	91.2 %	10.4 %	8.7 %
Imipenem (134, 114)	0.25	0.25	1	1	94.0 %	95.6 %	5.9 %	4.3 %
Imipenem/relebactam (134, 114)	≤0.12	0.25	0.25	0.25	100.0 %	97.4 %	0.0 %	2.6 %
Meropenem (134, 114)	≤0.12	≤0.12	0.5	≤0.12	98.5 %	97.4 %	1.4 %	2.6 %
Piperacillin/tazobactam (134, 114)	4	≤4	>64	32	64.9 %	80.7 %	35.0 %	19.3 %
**ESBL - producing *K. pneumoniae***								
Amikacin (204, 324)	≤ 8	≤ 8	32	>32	81.8 %	69.7 %	18.1 %	30.2 %
Aztreonam (204,	>8	>8	>16	>8	5.9 %	4.0 %	94.1 %	95.9 %
Cefepime (204, 324)	>16	>16	>16	>16	9.8 %	6.1 %	90.2 %	93.8 %
Ceftazidime (204, 324)	>16	>16	>16	>16	6.4 %	5.8 %	93.6 %	94.1 %
Ceftazidime/avibactam (204, 324)	0.5	1	2	>16	94.1 %	87.9 %	5.8 %	12.0 %
Ceftolozane/tazobactam (204, 324)	>8	>16	>16	>16	18.1 %	20.3 %	81.8 %	79.6 %
Ceftriaxone (204, 324)	>8	>8	>8	>8	0.9 %	0.3 %	99.0 %	99.6 %
Ciprofloxacin (77, 0)	>2	‒	>2	‒	10.1 %	‒	89.6 %	‒
Colistin (204, 324)	≤1	≤1	>4	>4	81.9 %	75.3 %	18.1 %	24.6 %
Ertapenem (204, 324)	>4	>2	>4	>4	31.4 %	27.1 %	68.6 %	72.8 %
Imipenem (204, 324)	8	>16	>16	>16	47.5 %	33.3 %	52.4 %	66.6 %
Imipenem/relebactam (204, 324)	0.25	0.25	1	8	93.6 %	87.0 %	6.3 %	12.9 %
Meropenem (204, 324)	4	16	>16	>16	56.4 %	42.9 %	43.6 %	57.1 %
Piperacillin/tazobactam (204, 324)	>64	>64	>64	>64	8.8 %	9.8 %	91.1 %	90.1 %
***P. aeruginosa* non-susceptible to ceftazidime**								
Amikacin (288, 405)	≤4	≤4	>32	32	87.8 %	89.3 %	12.1 %	10.6 %
Aztreonam (288, 405)	8	8	>16	>16	78.4 %	79.7 %	21.5 %	20.2 %
Cefepime (288, 405)	4	4	32	32	71.8 %	79.0 %	28.1 %	20.9 %
Ceftazidime (288, 405)	4	4	>32	>32	70.8 %	79.2 %	29.1 %	20.7 %
Ceftazidime/avibactam (288, 405)	2	2	16	8	87.5 %	90.3 %	12.5 %	9.6 %
Ceftolozane/tazobactam (288, 405)	0.5	0.5	8	4	88.8 %	92.8 %	11.1 %	7.1 %
Ciprofloxacin (136,0)	≤0.25	‒	>2	‒	61.7 %	‒	38.2 %	‒
Colistin (288, 405)	≤1	1	≤1	1	99.31 %	100.0 %	0.6 %	0.0 %
Imipenem (288, 405)	2	4	32	32	63.8 %	66.9 %	36.1 %	33.0 %
Imipenem/relebactam (288, 405)	1	1	4	4	80.9 %	81.2 %	19.1 %	18.7 %
Meropenem (288, 405)	1	1	32	16	76.3 %	82.7 %	23.6 %	17.2 %
Piperacillin/tazobactam (288, 405)	8	8	>64	> 64	65.2 %	73.5 %	34.7 %	26.4 %
***P. aeruginosa* non-susceptible to Meropenem**								
Amikacin (100, 130)	≤4	8	>32	>32	76.0 %	73.8 %	24.0 %	26.1 %
Aztreonam (100, 130)	16	16	>16	>16	60.0 %	55.3 %	40.0 %	44.6 %
Cefepime (100, 130)	16	8	>32	>32	45.0 %	50.7 %	55.0 %	49.2 %
Ceftazidime (100, 130)	16	8	>32	>32	47.0 %	53.0 %	53.0 %	46.9 %
Ceftazidime/avibactam (100, 130)	8	4	>32	>32	70.0 %	73.8 %	30.0 %	26.1 %
Ceftolozane/tazobactam (100, 130)	1	1	>32	>32	73.0 %	78.4 %	27.0 %	21.5 %
Colistin (100, 130)	≤1	1	≤1	1	100.0 %	100.0 %	0.0 %	0.0 %
Imipenem (100, 130)	32	32	>32	>32	7.0 %	10.7 %	93.0 %	89.2 %
Imipenem/relebactam (100, 130)	4	4	>32	>32	46.0 %	43.0 %	54.0 %	56.9 %
Meropenem (100, 130)	16	16	>32	>32	32.0 %	46.1 %	68.0 %	53.8 %
Piperacillin/tazobactam (100, 130)	32	32	>64	>64	36.0 %	42.3 %	64.0 %	57.6 %

GNB, Gram-Negative Bacilli; N1, Number of isolates tested during 2018‒2019; N2, Number of isolates tested during 2020‒2021; EUCAST, European Committee on Antimicrobial Susceptibility Testing; SI +SIE, Susceptible + Susceptible Increased exposure.

During the period 2018‒2019, among the *K. pneumoniae* evaluated in this study, the highest susceptibility rates were observed for ceftazidime/avibactam (MIC_50/90_, 0.5/2 µg/mL, *n* = 307, 96.2 % susceptible) and imipenem/relebactam (MIC_50/90_, 0.25/1 µg/mL, *n* = 306, 95.9 % susceptible). Ciprofloxacin, ceftriaxone and piperacillin/tazobactam showed poor *in vitro* activity against isolates of *K. pneumoniae* as displayed in Table 1. Similar results were observed during 2020‒2021 period for highest susceptibility rates, with ceftazidime/avibactam (MIC_50/90_, 0.5/4 µg/mL, *n* = 414, 91.4 % susceptible) and imipenem/relebactam (MIC_50/90_, 0.25/2 µg/mL, *n* = 411, 90.7 % susceptible) (Table 1).

*K. pneumoniae* exhibiting an ESBL phenotype was highly susceptible to ceftazidime/avibactam (MIC_50/90_, 0.25/2 µg/mL, *n* = 192, 94.1 %) and imipenem/relebactam (MIC_50/90_, 0.25/1 µg/mL, *n* = 191, 93.6 %) during the period 2018‒2019, as shown in Table 2. For the period 2020‒2021, similarly highest susceptibility rates were observed for ceftazidime/avibactam (MIC_50/90_, 1/ > 16 µg/mL, *n* = 285, 88.0 %) and imipenem/relebactam (MIC_50/90_, 0.25/8 µg/mL, *n* = 282, 87.0 %).

Further, colistin (MIC_50/90_, ≤ 1 µg/mL for both) and ceftolozane/tazobactam (MIC_50/90_, 0.5/8 µg/mL) were the most active *in vitro* agents tested against the *P. aeruginosa* isolates with susceptibility of 99.3 % (*n* = 286) and 88.9 % (*n* = 256) respectively during the period 2018‒2019. During the 2020‒2021 period, colistin (MIC_50/90_, 1 µg/mL for both), ceftolozane/tazobactam (MIC_50/90_, 0.5/4 µg/mL) and ceftazidime/avibactam (MIC_50/90_, 2/8 µg/mL) were the most active agents against the *P. aeruginosa* isolates with susceptibility of 100 % (*n* = 405), 92.8 % (*n* = 376) and 90.4 % (*n* = 366) respectively (Table 1). Further for *P. aeruginosa* isolates non-susceptible to meropenem, colistin (MIC_50/90_, ≤ 1/1 µg/mL) had high susceptibility rate of 100 % in both the study periods. Additionally, ceftolozane/tazobactam emerged as the most potent beta-lactam agent, demonstrating a susceptibility rate of 73 % in the 2018‒2019 period (*n* = 100) and 78 % in the 2020‒2021 period (*n* = 130) (Table 2).

### Detection of beta-lactamase encoding genes

The distribution of β-lactamase encoding genes according to bacterial species is shown in Table 3. 250 *E. coli* were tested, of these 17 were carbapenemases producers and 18 were ESBL producers. The carbapenamase producing isolates showed predominance of bla_KPC-2_ (*n* = 15; 88.2 %). bla_CTX__−__M-1–_240 G (*n* = 9, 50 %) was found in the majority of ESBL producing isolates.

**Table 3:** Distribution of beta-lactamase encoding genes according to bacterial species.

Bacterial species/B-lactamase encoding genes	Number	Percentage
*E. coli*	250^a^	‒
Carbapenemases	17	‒
*bla*_KPC-2_	15	88.2
*bla*_NDM-1_	2	11.8
ESBL	18	
*bla*_CTX−M-1–240G_	9	50.0
*bla*_CTX−M-8–240D_	4	22.2
*bla*_CMY-2-TYPE_	1	5.6
*bla*_CTX−M-15_	1	5.6
*bla*_CTX−M-2_	1	5.6
*bla*_CTX−M-2–240D_	1	5.6
*bla*_CTX−M-8_	1	5.6
*bla*_CTX−M-9–240D_	1	5.6
*bla*_CTX−M-9–240G_	1	5.6
*K. pneumoniae*	494^b^	‒
Carbapenemases	302	‒
*bla*_KPC-2_	260	86.1
*bla*_NDM-1_	37	12.3
*bla*_KPC-3_	7	2.3
*bla*_NDM-7_	6	2.0
*bla*_OXA-370_	3	1.0
*bla*_KPC-30_	1	0.3
*bla*_KPC-31_	1	0.3
*bla*_NDM-5_	1	0.3
*bla*_VIM-1_	1	0.3
ESBL	320	‒
*bla*_CTX−M-1–240G_	173	54.1
*bla*_CTX−M-2–240D_	53	16.6
*bla*_CTX−M-9–240D_	48	15.0
*bla*_CTX−M-15_	33	10.3
*bla*_CTX−M-14_	7	2.2
*bla*_CTX−M-2_	4	1.3
*bla*_CTX−M-8_	4	1.3
*bla*_CTX−M-8–240D_	3	0.9
*bla*_OXA-370_	3	0.9
*bla*_SHV-ESBL_	3	0.9
*bla*_CTX−M-9-TYPE_	2	0.6
*bla*_CTX−M-1–240D_	1	0.3
*bla*_CTX−M-9–240G_	1	0.3
*P. aeruginosa*	353^c^	‒
Carbapenemases	23	‒
*bla*_SPM-1_	10	43.5
*bla*_VIM-2_	9	39.1
*bla*_KPC-2_	6	26.1
*bla*_IMP-56_	1	4.3
ESBL	9	–
*bla*_CTX−M-2_	4	44.4
*bla*_CTX−M-229_	4	44.4
*bla*_GES-1_	1	11.1
Other mechanisms	22	95.7

a 250 *E. coli* were tested.

b 494 *K. pneumoniae* were tested.

c 353 *P. aeruginosa* were tested.

Among *K. pneumoniae*, 494 were tested which included carbapenamase (*n* = 302, 48.5 %) and ESBL (*n* = 320, 51.4 %) producers. Most of the isolates encoding carbapenamases were harboring *bla*_KPC-2_ (*n* = 260, 86.1 %) gene.

Among 353 of tested *P. aeruginosa*, 23 isolates had carbapenamases encoding genes and nine had ESBL encoding genes. *bla*_SPM-1_ (*n* = 10, 43.5 %) was most frequently detected in the carbapenamase group and *bla*_CTX__−__M-2_ and *bla*_CTX__−__M-229_ (*n* = 4, 44.4 % for both) in the ESBL group.

## Discussion

Surveillance studies play a crucial role in addressing the worldwide dissemination of AMR. These studies aid in comprehending the extent of the problem, unraveling the mechanisms underlying resistance, and generating data to facilitate the development of novel agents or enhance existing agents.^27^^,^^28^ Empirical regimens to treat GNB infections are based on the most prevalent pathogens causing infection and their antimicrobial susceptibility patterns. The WHO has also issued a public health warning and called nations to share AMR status through the implementation of the Global Antimicrobial Resistance Surveillance System (GLASS). In 2018, Brazil initiated its national antimicrobial surveillance program (BR-GLASS). However, considering the observed rise in resistance following the pandemic, it is imperative to encourage additional surveillance in Brazil, to gain greater comprehension of the present situation.^29^^,^^30^ In the current study, we assessed the AMR trends for two time periods, 2018‒2019 and 2020‒2021, to assess the impact of COVID-19 on the antimicrobial status.

In our study the most frequent GNB isolated from the RTI, UTI and IAI sites included *E. coli, K. pneumoniae* and *P. aeruginosa.* In the previous SMART study done in Brazil (2016‒2017), ceftolozane/tazobactam had shown high antibacterial *in vitro* activity against *E. coli* with > 90 % susceptibility.^7^ Similar results were observed in the current extended study during both the time periods (96.0 % for 2018‒2019 and 98.5 % for 2020‒2021). Comparable susceptibility profiles have been documented in prior research conducted in Eastern and Western Europe, Portugal, the United States, Hong Kong, and South Korea.^23^^,^^31^, ^32^, ^33^ GNB isolates collected from ICU in 7 different Asian countries in another SMART study (2017‒2019) showed ceftolozane/tazobactam having 86 % susceptibility for *E. coli*.^23^ The STEP multicenter study in Portugal also reported high susceptibility for *E. coli* (99.4 %) among ICU patients.^31^ Similar results were observed in a study (2012‒2018) including various European countries with ceftolozane/tazobactam demonstrating potent *in vitro* activity against *E. coli* from both Western Europe (99 %) and Eastern Europe (96 %).^33^ Despite the lack of clarity regarding the precise role of ceftolozane/tazobactam against ESBL-producing organisms, it has demonstrated promising outcomes in the treatment of ESBL-producing *Enterobacterales*, including severe infections.^16^^,^^34^, ^35^, ^36^ In a multicenter, retrospective study conducted in Italy, favorable clinical outcomes were observed in 84 % of patients with severe infections caused by ESBL-producing *Enterobacterales* who were treated with ceftolozane/tazobactam.^35^ A pooled analysis of Phase 3 clinical trials reported around 72 % of ESBL-producing Enterobacteriaceae (88 % for *E. coli* and 36 % for *K. pneumoniae*) susceptible to ceftolozane/tazobactam.^36^ Many of the global surveillance studies also demonstrated high susceptibility of ESBL producing *Enterobacterales* to ceftolozane/tazobactam for critically-ill and immunocompromised patients.^34^^,^^37^ Consistent with these, in our study also, the combination was susceptible for ESBL-producing *E. coli* in both study time periods, 84.3 % during 2018‒2019 and 93.8 % during 2020‒2021, similar to previous SMART study in Brazil, emphasizing it as an alternative treatment therapy for ESBL-producing organisms.^19^

Significant rates of ciprofloxacin resistance (> 50 %) were detected in *E. coli* and *K. pneumoniae* isolates during the 2018‒2019 study period, validated by the findings of SMART Brazil – 2016‒2018 that disfavor the empirical prescribing of this fluoroquinolone in our specific setting.^19^

Among the three groups studied*, K. pneumoniae* showed higher resistance to many treatment drugs. The resistance rates to ceftolozane/tazobactam, ceftriaxone and piperacillin/tazobactam were high (> 50 %) for both time periods studied. Similar resistance rates for ceftolozane/tazobactam were observed in some previous studies in Saudi Arabia (51.6 %), Poland (70 %) and Asian-Pacific (APAC) countries (43.4 % for ESBL non-CRE isolates).^38^, ^39^, ^40^ However, it is important to highlight that this present study had a limitation and was unable to identify if any of the ESBLs were also CRE isolates, which may justify the susceptibility of these isolates to those B-lactam.

There have been increased occurrences of carbapenem resistance in strains of *P. aeruginosa*, reported as over 60 % in Brazilian hospitals, causing high mortality.^41^^,^^42^ Mutations affecting the permeability of the microorganisms to carbapenems, and further overexpression of efflux systems might be one of the major non-enzymatic resistance mechanisms. However, it is worth noting that imipenem is not subject to efflux in *P. aeruginosa*. Based on the findings of numerous investigations, ceftolozane/tazobactam is a highly efficacious agent against *P. aerugin*osa isolates.^39^, ^40^, ^41^, ^42^ One of the studies from Poland reported that 86.0 % of carbapenem-resistant P. aeruginosa were susceptible to ceftolozane/tazobactam.^39^ Pfaller et al. conducted a study in 7 APAC countries and reported ceftolozane/tazobactam as the most potent against *P. aeruginosa* isolates with 90.8 % susceptibility.^40^ Similar results were reported in our study, ceftolozane/tazobactam showed high susceptibility against *P. aeruginosa* isolates in 2018‒2019 (88.8 %) and 2020‒2021 (92.8 %). It also demonstrated good susceptibility for *P. aeruginosa* isolates non-susceptible to ceftazidime and meropenem.

In this current study, *bla*_KPC-2_ gene was found in only six isolates of *P. aeruginosa.* This finding is consistent with earlier studies reporting a low prevalence of carbapenemase production.^41^^,^^43^^,^^44^ In the present study, *bla*_KPC-2_ was identified as the most prevalent carbapenemase encoding gene among *Enterobacterales* species. Additionally, the presence of *bla*_KPC-3_ and *bla*_KPC-30_, which have been infrequently reported in Brazil, was also observed. Surveillance studies thus help provide more understanding and opportunities to find better treatment options for resistant strains.

The COVID-19 pandemic has resulted in a surge of hospitalizations and ICU admissions, leading to a significant escalation in antibiotic usage, which has further accelerated the spread of AMR. Consequently, it is imperative to conduct a comprehensive evaluation of AMR in the post-COVID-19 era. This is particularly crucial in Brazilian hospitals, where GNB infections are highly prevalent, and the exacerbation of the AMR problem during the pandemic has been observed.^30^^,^^45^^,^^46^ In the present study, we observed increased resistance (> 10 %) of many antibiotics for *K. pneumoniae* including imipenem (14.1 %), meropenem (12.9 %) and amikacin (10.2 %) before (2018‒2019) and after pandemic (2020‒2021). Consequently, it is imperative to focus on developing novel antibiotic therapies, preventing the excessive use of existing medications, and placing significant emphasis on surveillance studies to get a comprehensive understanding of the present situation.

## Conclusion

This study demonstrates the favorable *in vitro* activity of ceftolozane/tazobactam against different GNB infections in Brazil. Our findings indicate that ceftolozane/tazobactam exhibits potent *in vitro* activity against *E. coli* and *P. aeruginosa* isolates. However, it is important to note that this antibiotic showed limited *in vitro* activity against *K. pneumoniae* in the Brazilian cohort, likely due to the widespread production of ESBL and *bla*_KPC-2_. As new mechanisms of antibiotic resistance continue to emerge, especially with the rise of carbapenem resistance, it is crucial to assess the current landscape of antimicrobial resistance to identify optimal therapeutic approaches.

Ceftolozane/tazobactam demonstrates significant *in vitro* susceptibility against carbapenem-resistant *P. aeruginosa*, which positions it as an important treatment option. Further studies are warranted to enhance our understanding of the treatment options available for multidrug-resistant organisms causing infections and to prevent unfavourable outcomes in patients.

## Funding

MSD Brazil, a subsidiary of Merck & Co., Inc., Rahway, NJ, USA, provided financial support for this study.

## Conflicts of interest

Amanda Azevedo Bittencourt, Vinicius Lima Faustino, Paula de Mendonça Batista, Marina Della Negra de Paula and Thales José Polis are employees of MSD subsidiaries of Merck & Co., Inc., Rahway, NJ, USA. Lays Paulino Leonel is employee of IQVIA Brazil.
